# Hypermethylation and reduced expression of *Gtl2*, *Rian* and *Mirg* at the *Dlk1-Dio3* imprinted locus as a marker for poor developmental potential of mouse embryonic stem cells

**DOI:** 10.1016/j.scr.2020.101931

**Published:** 2020-10

**Authors:** Maria Schacker, Yi-Han Cheng, Melanie Eckersley-Maslin, Richard Michael Snaith, William Henry Colledge

**Affiliations:** a Reproductive Physiology Group, Physiology, Development and Neuroscience, University of Cambridge, Downing Street, Cambridge CB2 3EG, UK; b Medimmune, Sir Aaron Klug Building, Granta Park, Cambridge CB21 6GH, UK; c Epigenetics Programme, Babraham Institute, Cambridge CB22 3AT, UK

**Keywords:** Embryonic stem cells, Epigenomics, Gene expression, Embryonic development, Methylation, Imprinting

## Abstract

Mouse embryonic stem cells (ESCs) have played a crucial role in biomedical research where they can be used to elucidate gene function through the generation of genetically modified mice. A critical requirement for the success of this technology is the ability of ESCs to contribute to viable chimaeras with germ-line transmission of the genetically modified allele. We have identified several ESC clones that cause embryonic death of chimaeras at mid to late gestation stages. These clones had a normal karyotype, were pathogen free and their *in vitro* differentiation potential was not compromised. Chimaeric embryos developed normally up to E13.5 but showed a significant decrease in embryo survival by E17.5 with frequent haemorrhaging. We investigated the relationship between the ESCs transcriptional and epigenomic state and their ability to contribute to viable chimaeras. RNA sequencing identified four genes (*Gtl2, Rian, Mirg* and *Rtl1as*) located in the *Dlk1-Dio3* imprinted locus that were expressed at lower levels in the compromised ESC clones and this was confirmed by qRT-PCR. Bisulphite sequencing analysis showed significant hypermethylation at the *Dlk1-Dio3* imprinted locus with no consistent differences in methylation patterns at other imprinted loci. Treatment of the compromised ESCs with 5-azacytidine reactivated stable expression of *Gtl2* and rescued the lethal phenotype but only gave low level chimaeras.

## Highlights

• Reduced expression of *Gtl2*, *Rian* and *Mirg* in ES cells compromised survival of chimaeric fetuses.
• Reduced expression of *Gtl2*, *Rian* and *Mirg* was associated with hypermethylation.
• Compromised ES cells can be identified and eliminated from experiments to reduce animal use.

## Graphical abstract

**Figure:**
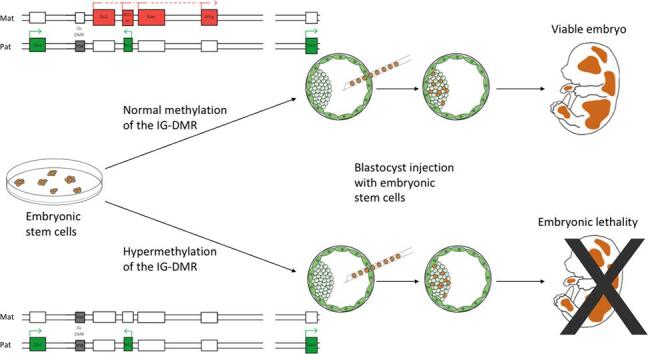

## 1 Introduction

Mouse embryonic stem cells (ESCs) have been a powerful tool to study mammalian gene function *in vivo*. ESCs can be used to recapitulate and study developmental mechanisms and diseases *in vivo* due to their ability to differentiate into cells of the three embryonic germ layers and contribute to all tissues of the developing embryo (Bradley et al., 1984). ESCs can be genetically manipulated in culture and injected into wild-type blastocysts where they combine with the inner cell mass to produce chimaeric offspring with potential germline transmission of the genetic alteration (Thompson et al., 1989). This method has been widely used to produce gene-targeted mice to study the role and function of the gene of interest. While CRISPR-Cas9-mediated gene editing in zygotes is increasingly used to generate transgenic mice, ESCs still have a role to play by allowing screening in culture to identify complex gene modifications that may be too inefficient or complex for zygote manipulation and to eliminate the need to generate many founder mice. In addition, there is a considerable pre-existing resource of gene targeted ESCs that have been produced by the Gene Targetting Consortium that have not yet been converted into mice.

During culture however, some ESC lines lose their ability to give rise to high contribution chimaeras. It is known that factors such as an abnormal karyotype can have an effect on whether or not cells contribute to the developing embryo (Longo et al., 1997). However, sometimes ESCs contribute to the inner cell mass but the developing embryo dies at a later stage during gestation for no obvious reason. We have identified a number of such ESC clones with compromised developmental potential and investigated the potential existence of a genetic or epigenetic marker common to all these ESC clones that could serve as a predictor for the developmental potency of ESCs.

It has been shown that iPSCs that fail to give rise to viable high percentage chimaeras do not express the maternally expressed, imprinted genes *Gtl2*, *Rian* and *Mirg*, all located in the *Dlk1-Dio3* imprinted locus on chromosome 12 (Stadtfeld et al., 2010). Here we show that in mouse ESCs, there is also a relationship between the expression levels of the maternally expressed genes from the *Dlk1-Dio3* imprinted locus and the ability of the ESCs to contribute to viable chimaeras.

## 2 Materials and methods

### 2.1 ESC clones

All the ESCs used in this study are sub-clones from the parental male ES cell line ESC(Ctrl)1, which was derived from blastocysts from a C57BL/6JOlaHsd female × 129S6/NTac male cross. This means that the X-chromosome present in all the ESCs is derived from C57BL/6JOla Hsd mice. All other ESCs (ESC(Ctrl)2–7 and ESC(Comp)1–7) in this study have come from a sequential gene targeting project (unpublished data), meaning that multiple site-specific mutations (none of which are known to be implicated in or necessary for embryonic development) were introduced into the cells. All the ESC clones are therefore sub-clones that have gone through between 4 and 6 sequential gene targeting steps starting from the ESC(Ctrl)1 parental cell line.

### 2.2 Mouse ESC culture

ESCs were cultured on mitotically inactivated mouse embryonic fibroblasts (MEFs) in standard ESC medium (DMEM (Invitrogen) with 15% FCS (PAN Biotech), 4 mM GlutaMax (Gibco), 1X non essential amino acids (Gibco), 20 mM Hepes buffer (Gibco), 0.1 mM β-mercaptoethanol (Gibco), 1.5 × 10^3^ U/ml LIF (Millipore) in a 37 °C humidified incubator with 5% CO_2_. When 70–80% confluent, the cells were passaged. The ESCs did not exceed passage 20. For *Gtl2* rescue experiments, ESCs were cultured feeder-free on gelatin-coated plates in 2i (Ying et al., 2008) media (with Vitamin A) or EPSC media (Yang et al., 2017), or on MEFs in standard ESC media supplemented with 0.5 μM 5-azacytidine for 48 h. Longer incubation in 5-azacytidine resulted in cell death.

### 2.3 Cell labelling

ESCs were treated with trypsin and plated at a density of 5 × 10^6^ cells per 10 cm MEF plate 24 h prior to the transfection. The vectors were used at the concentration of 2 ng of pPBCAG-Venus-IP and pPBCAG-H2BtdTomato-IH and 1 ng of CAGG-PBase (pCyL43) per 5x10^6^ cells. All vectors were kindly supplied by Professor Azim Surani (The Gurdon Institute, University of Cambridge). Nucleofection was carried out using the Mouse ES Cell Nucleofector Kit (Lonza). 5 × 10^6^ cells were re-suspended in 90 ul Mouse ES Nucleofector Solution and mixed with the targeting vectors, which were diluted in 10 ul Mouse ES Nucleofector Solution. The cells were nucleofected using the A-013 program and then seeded onto MEF plates at 5 × 10^6^ cells per plate. After 24 h without selection, the medium was replaced with fresh ESC medium containing the appropriate selection agent (1.75 ug/ml Puromycin or 150 ug/ml Hygromycin). Cells were selected for 9 days with the medium changed every 1–2 days.

### 2.4 RNA sequencing

#### 2.4.1 Sample preparation

RNA was extracted from three biological replicates of the original ESC clones (ESC(Ctrl)1–3 and ESC(Comp)1–4) that were found to have different developmental potential after blastocyst injections and analysis of chimaeras. Cells were passaged twice after thawing to remove feeder cells before RNA extraction. The ESCs used for RNA sequencing were of low passages (p5 – p8).

#### 2.4.2 RNA-seq library preparation and sequencing

Library preparation was performed using the Illumina TruSeqHT kit according to the manufacturer’s protocol. Single-end sequencing was performed and each sample was sequenced in four lanes on a NextSeq500 (Illumina) instrument in the same High-Output 75 cycle single-end sequencing run. Sequencing depth was approximately 10 M reads per sample. Library preparation and sequencing was performed by the Cambridge Genomics Service (Department of Pathology, University of Cambridge, UK).

#### 2.4.3 Data analysis

The RNAseq data has been deposited on the NCBI Gene Expression Omnibus database (accession number GSE149628). Mapping of sequence reads and statistical analysis was performed using the free bioinformatics platform Galaxy (www.usegalaxy.org/).

After concatenating the sequencing output files from the four lanes for each sample, the quality of sequenced reads was assessed with FastQC (www.bioinformatics.babraham.ac.uk/projects/fastqc/) to analyse basic statistics such as per base sequence quality, per sequence quality scores, per base sequence content, sequence length distribution, Kmer content and adapter content. Reads were trimmed using Fastx Trimmer by 8 and 4 bases at the start and end of each read respectively to improve quality. TopHat2 Version 0.9 with default settings was used to align reads to the mouse genome (GRCm38/mm10). Reads that mapped uniquely were used for downstream analysis. htseq-count Version 0.6.1galaxy1 was applied to count the number of reads mapping to each gene. Intersection-strict mode was chosen to exclude reads that overlap more than one gene or reads that do not intersect a given gene with each read position. The Gencode release M7 was used to annotate the genome. Differential expression analysis was subsequently performed with DESeq2 Version 2.1.8.3 which uses a negative binomial distribution and a mean dependent local regression model. Results were considered significant where the P-value was <0.05.

The web based Database for Annotation, Visualization and Integrated Discovery (DAVID) Functional Annotation Tool (https://david.ncifcrf.gov/summary.jsp) was used to perform functional annotations of the list of significantly up- and downregulated genes. Gene ontology (GO) term (biological processes) and Kyoto Encyclopedia of Genes and Genomes (KEGG) pathway enrichment as well as UniProt tissue term over-representation were analysed.

### 2.5 Methylation analysis (Eckersley-Maslin et al., 2016)

#### 2.5.1 Sample and library preparation

DNA methylation of imprinted regions was analysed using the IMPLICON method (Klobučar et al., 2020) Genomic DNA (gDNA) was extracted from three biological replicates of the original seven ESC clones used for RNAseq analysis plus an additional seven clones (ESC(Ctrl)1–7 and ESC(Comp)1–7). Cells were passaged twice after thawing before gDNA extraction to remove the feeder cells. The ESCs used for methylation analysis were of low passages (p5 - p8). 1 ug of DNA was bisulfite converted using the EZ DNA Methylation Kit (Zymo Research, D5020), according to the manufacturer’s instructions. Samples were eluted in 66 ul elution buffer to get a concentration of 15 ng/ul. For each sample, each imprinted region was amplified in a separate PCR reaction and then pooled. 1.5X AMPure beads were used for PCR clean up. In a second PCR reaction, barcoded adapters were attached to the samples for sequencing. 1X AMPure beads were used for PCR clean up. Libraries were sequenced as paired-end 150 bp reads on a MiSeq Instrument. 10% PhIX spike-in was included due to the low-complexity libraries. Primer sequences available on request (unpublished methods).

#### 2.5.2 Methylation data analysis

In brief, poor quality base calls and adapters were trimmed. Using Bismark (www.bioinformatics.babraham.ac.uk/projects/bismark/) the sequences were subsequently aligned to the mouse genome (GRCm38/mm10) and deduplication was performed to remove PCR bias. Methylation data were quantified using Seqmonk (www.bioinformatics.babraham.ac.uk/projects/seqmonk/) and probes were defined for every CpG that was included in the amplicons. Percentage methylation was calculated for each of the probes that had at least 30 reads. The overall percentage methylation for each imprinted region was calculated as the mean of individual CpG sites. For CpG methylation levels, a homoscedastic two-tailed *t*-test was used to determine significance between at least 3 biological replicates for each sample. Results were considered significant where the P-value was < 0.05.

### 2.6 qPCR gene expression analysis

Quantitative PCR (qPCR) was performed using qPCRBIO SyGreen Mix Lo-ROX (PCR Biosystems) on the 7500 Fast Real-Time PCR System (Applied Biosystems). Components of the reaction mix and thermal cycling conditions are outlined in Supplementary Table S1. All primers were designed using the NCBI Primer-Blast tool and are listed in Supplementary Table S2. The amplification efficiency of primer pairs was assessed using serial dilutions to generate a standard curve. Relative fold-changes were calculated using the delta-delta-C_t_ method (Livak and Schmittgen, 2001). Appropriate statistical analyses were performed using GraphPad Prism and results were considered significant where the P-value was <0.05.

### 2.7 Blastocyst injections

#### 2.7.1 Animals

Timed matings of C57BL/6NCrl mice (Charles River, UK) were set up to provide blastocysts at 3.5 dpc (days post coitum) for ESC injections. 2.5 dpc pseudopregnant F1 females (C57BL/6 × CBA) for embryo transfer were derived by mating with vasectomised males. Experimental procedures involving mice were performed under the authority of a United Kingdom Home Office Project License and approved by the Cambridge University Biomedical Services Local Ethics Committee.

#### 2.7.2 ESC injections and embryo transfers

ESC medium was replaced on the cells 1–2 h prior to the injections. The cells were then trypsinised and resuspended in a small volume of ESC media. Using a Leitz Micromanipulator, 10–12 ESCs were injected into each blastocyst. After 2–3 h of recovery to give them time to re-expand, the injected blastocysts were transferred into the 2.5 dpc pseudopregnant F1 female recipients using a NSET (non surgical embryo transfer) device (Green et al., 2009).

#### 2.7.3 Analysis of chimaeras

In the first instance, pregnant females were weighed at gestational days e10.5, e13.5, e15.5. and e17.5 to monitor pregnancy status. Chimaerism of pups born was assessed by coat colour. Later, pregnant females were sacrificed by dislocation of the neck at e13.5 or e17.5 to define the embryonic phenotype. The embryos were imaged using both the Zeiss SteREO Discover.V8 Stereomicroscope and the Leica M205 FA fluorescence microscope with a DFC7000T camera. To analyse ESC contribution, the grey-scale images of e17.5 chimaeric (fluorescent) embryos were analysed in ImageJ. Their outline was drawn and the mean grey value of the defined area was measured.

#### 2.7.4 Histology

Embryonic tissues were fixed in 4% paraformaldehyde (PFA) in buffered saline for 5 h and processed for histological sectioning in wax blocks. Tissue sections were cut at 7–8 um using a microtome, mounted on glass slides and stained with haematoxylin and eosin. Slides were imaged using a NanoZoomer digital slide scanner and images were analysed using the NDP.view2 software. Measurements of vessel diameters (of both intact and disrupted vessels) were performed blinded and the Mann-Whitney *U* test was performed to compare vessel diameter between the two groups.

## 3 Results

### 3.1 Compromised ESCs cause late gestation embryonic lethality

Several ESC clones with compromised developmental potential were identified during a sequential gene targeting project. These ESC clones failed to give rise to viable chimaeras when injected into E3.5 blastocyst stage embryos. It can be assumed, that the gene-targeting event per se is not the cause of the phenotype in these clones, because the targeted genes are not involved in embryonic development and do not alter the developmental potential of the ESCs as shown by genetically equivalent targeted clones not being compromised. Specifically, for each gene targeting event, a large number of ESC clones were selected, and, of those, several ESC clones were selected for blastocyst injections. Typically, all selected clones give rise to high-percentage chimaeras. However, all of the compromised ESC clones (from this point onwards referred to as ESC (Comp) cells) used in this study repeatedly failed to give rise to viable chimaeras (Table 1) despite their sister-clones with the same targetted alteration producing large numbers of high-percentage chimaeras (unpublished data). Here, we have investigated the route cause for the compromised developmental potency of the ESC(Comp) clones. The ESCs were all male (XY), had a normal karyotype and were negative for pathogens (data not shown). To exclude the possibility that the blastocysts had simply not implanted into the uterus of the pseudopregnant recipient females, these were routinely weighed to monitor their pregnancy. Most females gained weight but then lost it during the second half of gestation, suggesting that they were pregnant but that the embryos died late in pregnancy. In comparison, high percentage chimaeras were obtained from all of the control cell clones (hereafter referred to as ESC(Ctrl) cells; Table 1). All cell clones used in this study are sub-clones of the same parental wild type (C57BL/6JOlaHsd × 129S6/NTac F1) ESC line, ESC(Ctrl) 1.

**Table 1:** Blastocyst injection data for ESC clones.

Cell line	Number of pups	Number of chimaeric Pups (%)
ESC(Ctrl) 1	Not applicable – parental ESC line used to derive all other targetted clones	Not applicable
ESC(Ctrl) 2	8	5 (62.5)
ESC(Ctrl) 3	4	3 (75)
ESC(Ctrl) 4	22	20 (91)
ESC(Ctrl) 5	18	18 (100)
ESC(Ctrl) 6	20	18 (90)
ESC(Ctrl) 7	14	14 (100)
ESC(Comp) 1	0	0
ESC(Comp) 2	0	0
ESC(Comp) 3	1	0 (0)
ESC(Comp) 4	0	0
ESC(Comp) 5	1	0 (0)
ESC(Comp) 6	0	0
ESC(Comp) 7	0	0

### 3.2 Phenotypic and histological analysis of compromised fetuses.

**Fig. 1:**
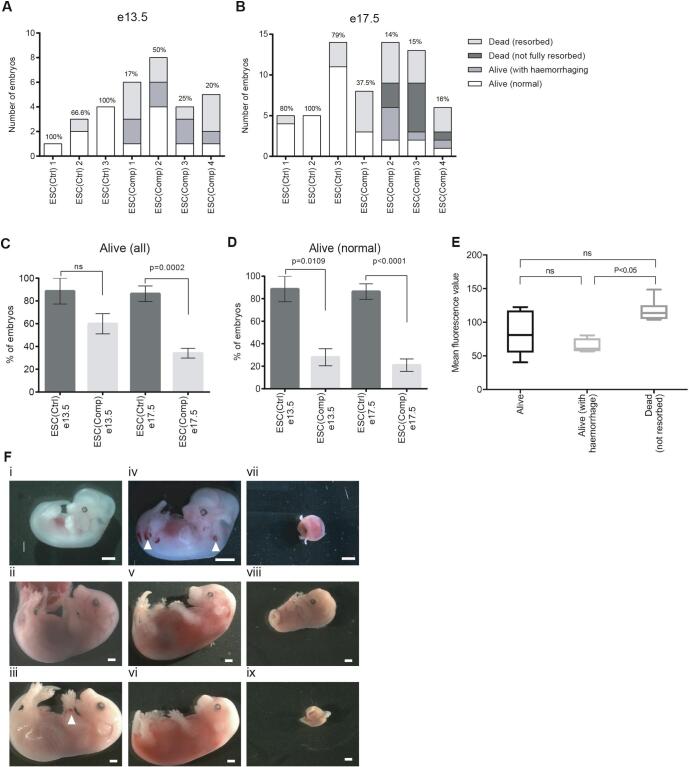
Embryonic lethal phenotype of ESC(Comp) chimaeras: (A-B) Phenotypes of chimaeric embryos at e13.5 and e17.5. Numbers above bars are the percentages of live (normal) embryos. (C-D) Percentages of live embryos compared between the ESC(Ctrl) and ESC(Comp) groups for both time points including all live embryos (anatomically normal), with haemorrhaging) (C) or live embryos (anatomically normal) with no haemorrhaging (D). P-values were calculated using Fisher’s exact test. (E) Quantification of ES cell contribution in chimaeras by fluorescence imaging. Box plots showing fluorescence per group with the line inside the box representing the median and whiskers ranging from the 10th to 90th percentile. P-value was calculated using a Mann-Whitney *U* test (p < 0.05). (F) Range of phenotypes: (i) live (normal) e13.5, (ii) live (normal) e17.5, (iii) live (haemorrhaging) e17.5, (iv) live (haemorrhaging) e13.5, (v,vi) dead (not resorbed) e17.5, (vii) resorption e13.5, (viii, ix) resorptions e17.5 (viii, ix). White arrow-heads indicate small foci of haemorrhaging. White scale bars = 1 mm.

To track the fate of the chimaeric embryos and to better define the lethal phenotype, the ESCs were fluorescently labelled using the PiggyBac transposase system (Supplementary Fig. S1). The phenotype of the chimaeric embryos was analysed at gestational days e13.5 and e17.5 using these labelled ESCs. Chimaeric embryos were identified by the presence of fluorescence. As shown in Fig. 1A and B, four main phenotypes were observed: live and healthy (normal), live with haemorrhaging, dead (not resorbed) and dead (resorbed). At e17.5, embryonic lethality was higher in the ESC(Comp) derived chimaeras while most of the remaining live embryos showed severe haemorrhaging (Fig. 1A, B). Overall, the number of live embryos was significantly reduced at e17.5 but not at e13.5 (Fig. 1C). However, looking at just the live (normal) embryos that did not show any haemorrhaging, this number was significantly reduced at both time points (Fig. 1D), indicating that although lethality seems to mostly occur later in gestation, the haemorrhaging phenotype starts to manifest as early as e13.5. Haemorrhaging was most obvious in the liver and the limbs (Fig. 1F). These data show that the number of abnormal and dead embryos is consistently higher in the compromised ESC group. While ESC contribution was grossly similar in all embryos (Supplementary Fig. S1), Fig. 1E indicates that there might be a correlation between the contribution of ESC(Comp) cells determined by ESC fluorescence quantification and the severity of the phenotype. Although we found a small number of live normal fetuses at e17.5 in the compromised ESC group, we rarely found that these ESCs gave rise to life pups (Table 1), suggesting that additional lethality might occur between e17.5 and birth.

**Fig. 2:**
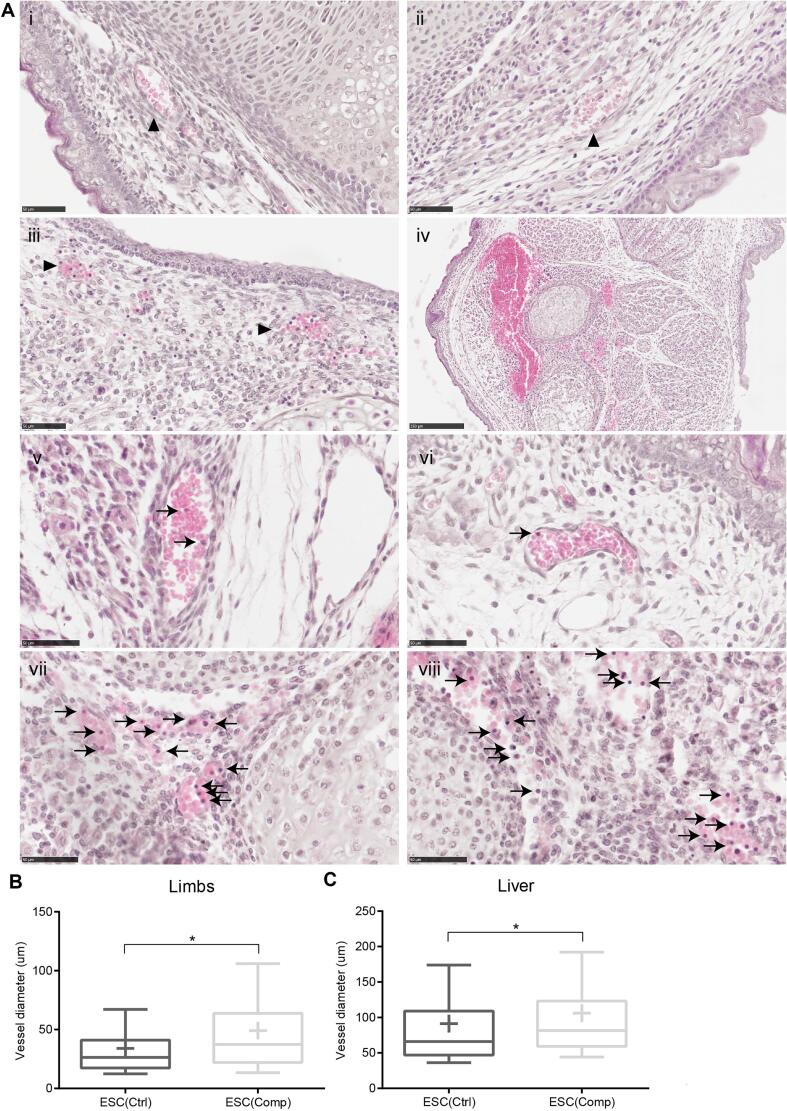
Histological analysis at e17.5: (A) Haematoxylin and eosin images of sections of the limbs. (i) ESC(Ctrl), (ii-iii) ESC(Comp). (iv) haemorrhage in ESC(Comp). (v-vi) ESC(Ctrl), (vii-viii) ESC(Comp). Arrows showing nucleated blood cells. Scale bar in (iv) is 250 um, all others are 50 um. (B-C) Box plots showing diameter of blood vessels in the limbs (B) and liver (C). Whiskers range from 10th to 90th percentile. The line inside the box represents the median, the mean is shown by a cross. Asterisk represent significant differences as calculated by the Mann-Whitney *U* test (p < 0.05).

To further analyse the haemorrhaging phenotype, blood vessels in the liver and the limbs were examined more closely by histology (Fig. 2A). It was noted that the blood vessel walls were less well defined in the ESC(Comp) chimaeras and it looked like the integrity of the epithelial vasculature was impaired (Fig. 2A, ii). Some ruptured vessels were also observed (Fig. 2A, iii). The severity of this phenotype varied from none or very few ruptured blood vessels to major haemorrhaging (Fig. 2A, iv), but this was not observed in any of the ESC(Ctrl) samples. Measurements of the blood vessels in the liver and the limbs also showed that those of the ESC(Comp) chimaeras had a greater average diameter than those of the ESC(Ctrl) chimaeras (Fig. 2B, C). Additionally, we noticed that the number of nucleated blood cells appeared to be higher in the ESC(Comp) chimaeras than in the ESC(Ctrl) chimaeras (Fig. 2A, v-viii). These are probably nucleated erythrocytes or lymphocytes.

### 3.3 Transcriptomics analysis reveals differential expression of genes from the *Dlk1-Dio3* region in the ESC(Comp) clones

**Fig. 3:**
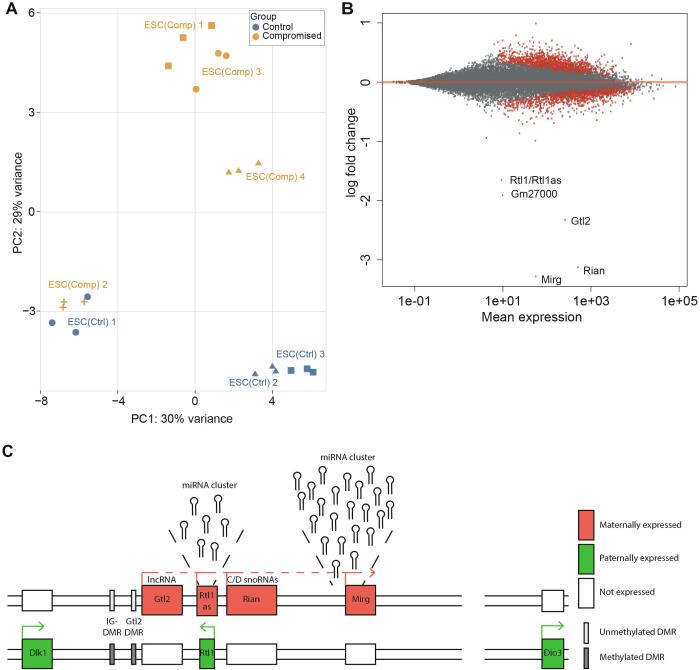
RNAseq shows downregulation of the maternal genes of the *Dlk1-Dio3* imprinted region in compromised clones: (A) PCA plot of RNAseq results. Identical shapes represent three biological replicates of the same cell line. Compromised clones are shown in orange, control clones in blue. (B) MA-plot comparing read counts of all control cell clones with read counts from all compromised cell clones. Differentially expressed genes are shown in red (p < 0.1, *t*-test with Benjamini Hochberg correction). (C) Schematic of the *Dlk1-Dio3* imprinted locus on mouse chromosome 12. (For interpretation of the references to colour in this figure legend, the reader is referred to the web version of this article.)

We hypothesised that the ESC(Comp) clones had undergone genetic or epigenetic changes during culture that interfere with their developmental potential during embryonic development. To identify genes that were differentially expressed between the ESC(Comp) and ESC(Ctrl) groups, we performed global transcriptomics analysis on a subset of clones using RNA sequencing. The ESC(Ctrl)1–3 and ESC(Comp)1–4 clones were the first ones identified from the blastocyst injection data that showed different developmental potentials so these were used for the RNAseq analysis. The ESC(Ctrl) and ESC(Comp) transcriptomes show a range of variation as illustrated in the principal component analysis (PCA) (Fig. 3A). While PCA does not show clear clustering of all the ESC(Ctrl) and ESC(Comp) clones, this probably reflects that these clones were all derived from a single parental ES cell line during a large sequential gene targeting project and are therefore not expected to have very variable gene expression profiles and any small gene expression differences are not captured very well by the PCA plot which includes a large number of genes. Overall, 251 genes were differentially expressed between ESC(Ctrl) and ESC(Comp) clones (p < 0.001; adjusted for multiple testing with the Benjamini-Hochberg procedure), of which 110 were downregulated and 141 were upregulated in the ESC(Comp) clones (Fig. 3B; Supplementary Table S3). Of particular interest were the five genes that were expressed differentially in the ESC(Comp) cells with a log_2_ fold change greater than −1.5 (Fig. 3B). Four out of these five genes (*Gtl2* (also known as *Meg3*), *Rian*, *Mirg* and *Rtl1/Rtl1as*) are located in the *Dlk1-Dio3* imprinted locus on chromosome 12 (Fig. 3C) and all showed lower expression in the compromised clones with log_2_ fold changes of −2.32, −3.12, 3.28 and −1.91, respectively.

### 3.4 Validation of RNAseq data

**Fig. 4:**
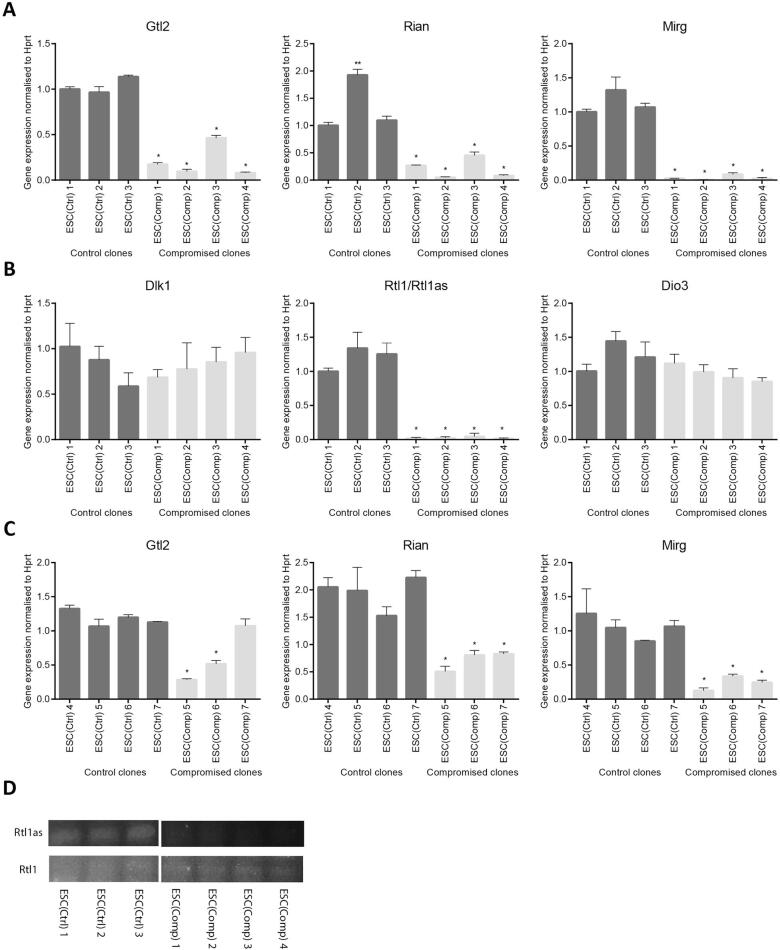
Validation of expression levels of genes from the *Dlk1-Dio3* imprinted locus: (A) qPCR validation of *Gtl2*, *Rian* and *Mirg* expression in ESC(Comp) clones compared to ESC(Ctrl) clones. (B) qPCR validation of *Dlk1*, *Rtl1/Rtl1as* and *Dio3* expression in ESC(Comp) clones compared to ESC(Ctrl) clones. (C) qPCR validation of *Gtl2*, *Rian* and *Mirg* expression in additional ESC(Comp) clones compared to ESC(Ctrl) clones (Error bars represent SEM. Stars represent significant differences as calculated by one-way ANOVA). (D) PCR after primer-specific reverse transcription showing expression levels of *Rtl1* and *Rtl1as*. ESC(Ctrl) and ESC(Comp) samples were run on the same gel, on non-adjacent lanes as indicated by white space between parts of the image.

To confirm the RNAseq data, the expression levels of transcripts associated with the *Dlk1*-*Dio3* imprinted locus were quantified by qRT-PCR (Fig. 4). The non-coding RNA transcript *Gtl2*, the small nucleolar RNA *Rian* and the miRNA-containing gene *Mirg* are all expressed from the maternal chromosome and were all lower in the ESC(Comp) clones (Fig. 4A). Expression of the *Rtl1 and Rtl1as* transcripts were also lower in the ESC(Comp) clones but the qRT-PCR method did not discriminate between the sense and the antisense transcripts (Fig. 4B).

To further confirm the relationship of the *Dlk1-Dio3* imprinted locus and the developmental potency of ESCs, the expression levels of the maternally expressed genes in this region were analysed in additional ESC(Ctrl) and ESC(Comp) clones (Fig. 4C). While the four additional ESC(Ctrl) clones all had expression levels similar to the ESC(Ctrl) clones tested initially, all three additional ESC(Comp) clones showed aberrant downregulation of *Rian* and *Mirg* and two of them also had significantly lower expression of *Gtl2*.

Interestingly, the relative expression of *Gtl2* (34%) in the compromised clones was higher than for *Rian* (25%) and *Mirg* (16%). This might be explained by expression of the non-imprinted Gm27000 pseudogene which is located on the X-chromosome of C57B/6 mice and has an identical sequence to the 3′ region of *Gtl2*. Next generation sequence analysis has confirmed that the X-chromosome in all the ES clones used in this study is from the C57Bl/6 strain. This might explain why the *Gtl2* levels are higher than the *Rian1* and *Mirg* levels in the compromised clones as the RNAseq data may pick up expression from the X-linked *Gtl2* pseudogene (Gm27000).

Since the paternally expressed *Rtl1*transcripts and the maternally expressed *Rtl1as* transcripts were not distinguishable by unstranded RNAseq and conventional qPCR, primer specific reverse transcription followed by PCR amplification was used to distinguish between these transcripts, as described previously (Labialle et al., 2008). This confirmed equal expression levels of *Rtl1* in all samples and reduction of *Rtl1as* expression in the ESC(Comp) clones (Fig. 4D) consistent with silencing of genes at the maternal locus.

### 3.5 Methylation analysis of IG-DMR and other imprinted regions

**Fig. 5:**
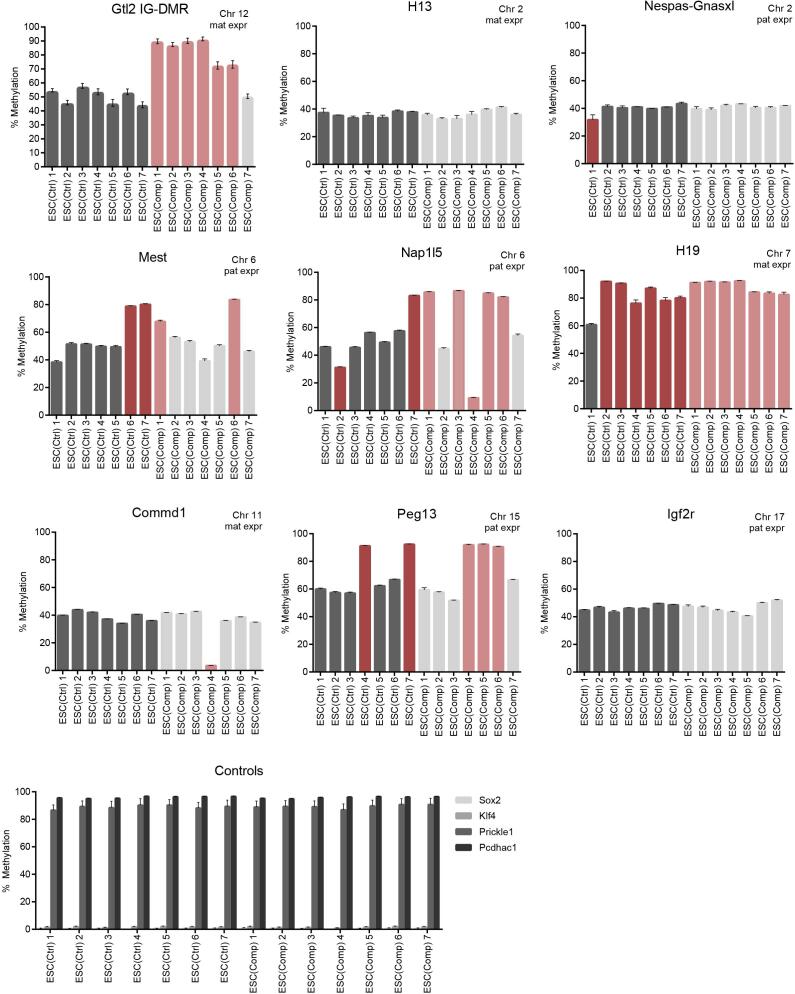
Methylation levels in differentially methylated regions of other imprinted genes in ESC(Ctrl) and ESC(Comp) clones. Methylation was measured using bisulfite sequencing. Red bars indicate aberrant methylation levels within each group (Ctrl or Comp). (For interpretation of the references to colour in this figure legend, the reader is referred to the web version of this article.)

The *Dlk1-Dio3* imprinted locus is controlled by several differentially methylated regions (DMRs) (Fig. 3C). The intergenic DMR (IG-DMR) located approximately 13 kb upstream of the *Gtl2* promoter has been shown to be the main regulator for expression of the maternal genes. These become methylated during spermatogenesis and are therefore methylated only on the paternal allele, but remain unmethylated on the maternal allele, hence allowing transcription of the maternal genes. We next determined whether abnormal methylation may be responsible for the differences in gene expression. Methylation levels of the IG-DMR were analysed using amplicon-based bisulfite sequencing. The ESC(Ctrl) clones all had methylation levels of approximately 50% which is expected for a germline imprinted region. Six of the seven ESC(Comp) clones, however, had significantly higher DNA methylation levels at the IG-DMR, consistent with gene expression patterns of those clones (Fig. 5). The ESC(Comp) 7 clone had normal methylation levels and this clone also had lower expression of *Rian* and *Mirg*, but not *Gtl2* (Fig. 4C). While it is possible that the *Gtl2* levels in this clone may be attributed to expression of the *Gtl2* pseudogene *Gm27000*, it is more likely that this clone has undergone downregulation of *Rian* and *Mirg* by a different mechanism.

DMRs of additional imprinted regions were also analysed (Fig. 5). These included both maternally methylated (Nespas-Gnasxl, Mest, Nap1l5, Peg13, Igf2r) and paternally methylated DMRs (H13, H19, Commd1) that are commonly examined in ESCs. Loss of imprinting (both hyper- and hypomethylation) was observed in some ESC(Ctrl) and ESC(Comp) clones, but no other region showed such a consistent difference in methylation levels between ESC(Ctrl) and ESC(Comp) clones as at the IG-DMR of the *Dlk1-Dio3* imprinted locus.

### 3.6 Methylation rescue

**Fig. 6:**
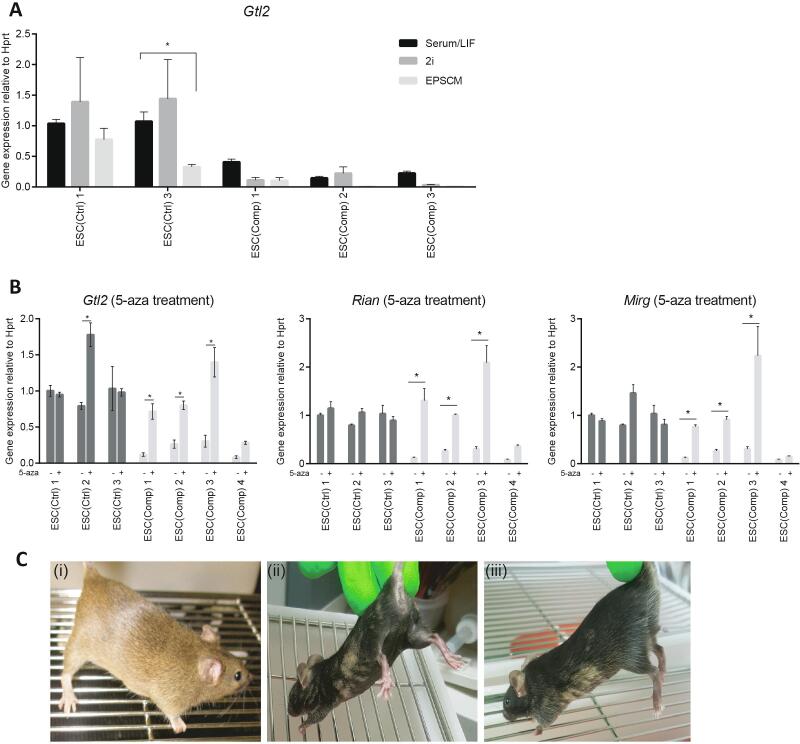
Gene expression analysis and expression rescue experiments. (A) *Gtl2* expression levels after culture in standard ES culture conditions (Serum/LIF), 2i and EPSCM media (Error bars represent SEM. Stars represent significant differences as calculated by two-way ANOVA). (B) *Gtl2*, *Rian* and *Mirg* expression levels before and after treatment with 0.5uM 5-azacytidine (Error bars represent SEM. Stars represent significant differences as calculated by one-way ANOVA). Expression of the house keeping gene *Hprt* was not altered by any of the treatments. (C) Chimaeras derived from (i) a non-compromised ESC clone or (ii and iii) 5-azacytidine treated ESCs. Photographs were taken at 2 months of age.

DNA methylation is a reversible DNA modification. Therefore, we reasoned that expression of *Gtl2* might be rescued by modifying the ESC culture conditions. Initially, we looked at the effects of culturing the cells in 2iLIF medium, which helps maintain ES cells in an undifferentiated ground state (Ying et al., 2008) or EPSC media, which helps maintain the differentiation potential of ES cells (Yang et al., 2017). Both of these are able to maintain the ESC state and they affect global DNA methylation levels (Ficz et al., 2013, Leitch et al., 2013, Yang et al., 2017). In particular, culture in both 2i media (Choi et al., 2017, Yagi et al., 2017) and EPSC media (unpublished data) has been shown to cause loss of DNA methylation. We cultured some of the cell clones in 2i and EPSCM for 3 and 5 passages respectively. However, overall these conditions did not have an effect on the expression levels of *Gtl2* in the ESC(Comp) cells (Fig. 6A). This was unsurprising as it is known that imprints are usually protected from the demethylation caused by 2i conditions (Ficz et al., 2013, Habibi et al., 2013).

The ESCs growing under our standard culture conditions were then treated with the DNA methyltransferase (DNMT) inhibitor 5-azacytidine, which is known to induce global loss of DNA methylation. 5-azacytidine treatment successfully rescued the expression of *Gtl2*, *Rian* and *Mirg* in some of the ESC(Comp) clones to levels similar of those in untreated ESC(Ctrl) clones. (Fig. 6B). Injection of those cells into blastocysts mainly gave rise to pups with no ESC contribution, but a small number of chimaeras were also born. However, these had extremely low ESC contribution as determined by coat colour (Fig. 6C ii-iii) compared to the parental non-compromised ES cell clones (Fig. 6C i). Test breeding of these low contribution chimaeras only gave rise to pups derived from the host blastocyst with no germline transmission of the injected ESCs (data not shown).

## 4 Discussion

We have found a correlation between down-regulation of the maternally expressed genes of the *Dlk1-Dio3* imprinted locus on mouse chromosome 12 and loss of the ability of ESCs to successfully contribute to viable chimeras. In particular, the compromised ESCs show reduced expression of *Gtl2, Rian, Mirg* and *Rtl1as*, a number of non-coding RNAs from the maternal chromosome. Consistent with these gene expression patterns, the locus is also hypermethylated in the intergenic differentially methylated region (IG-DMR). These effects are very similar to those found in induced pluripotent stem cells (iPSCs) where maintained expression of the maternally expressed genes from the *Dlk1-Dio3* imprinted locus is required for successful iPSC reprogramming and contribution to chimaeras (Stadtfeld et al., 2010).

The underlying mechanism for the hypermethylation and subsequent reduction in gene expression in the ESC(Comp) cells remains unclear. However, it is likely that the hypermethylation of the IG-DMR on the maternal allele in the ESC(Comp) clones is caused by epigenetic instability in ESCs that has been reported previously (Humpherys et al., 2001). It is known that *Dppa3* expression controls imprinting of the *Dlk1-Dio3* imprinted locus by antagonizing DNMT3A binding (Xu et al., 2015). However, we did not observe differential expression of *Dppa3* between the ESC(Ctrl) and ESC(Comp) cells (Supplementary Table S3). Furthermore, Stadtfeld et al. (2010) have also shown that reduced histone acetylation at the *Dlk1-Dio3* imprinted locus correlates with aberrant hypermethylation, and Das et al. (2015) reported that expression of the Polycomb Repression Complex 2 (*Prc2*) is required to maintain expression of *Gtl2, Rian,* and *Mirg* in mouse ESCs. ESCs with disrupted PRC2 activity lose expression of these genes due to DNA methylation at the IG-DMR. Interestingly, these gene expression changes specifically affect the maternally expressed genes of the *Dlk1-Dio3* imprinted locus since absence of an active PRC2 is not associated with any change in *Dlk1* expression, similar to what we find in our compromised ESCs. It would be interesting to examine the compromised ESCs for both reduced expression of *Prc2* and also reduced levels of histone acetylation.

It is also unclear whether the deregulated expression of *Gtl2, Rian,* and *Mirg* is the direct cause for the observed lethal phenotype or what might be the individual contribution of each transcript. Maternal deletion of *Gtl2-Rian-Mirg* expression results in perinatal death (Zhou et al., 2010), which is a less severe phenotype than we observe. In these mice however, increased expression of the paternal genes *Dlk1, Rtl1* and *Dio3* are found which might modulate the phenotype. The changes we observe between reduced *Gtl2/Mirg/Rian* expression levels and the detrimental phenotype of the ESCs are correlative and additional experiments involving temporal induction of *Gtl2/Mirg/Rian* expression would have to be performed to establish causality.

Some of the ESC(Comp) clones, but also the ESC(Ctrl) clones have changes in the methylation pattern of other imprinted genes. Particularly striking was the consistent hypermethylation of *H19* in all but the parental ES cell line. However, since loss of *H19* expression does not cause embryonic lethality (Leighton et al., 1995) this was not evaluated further. In fact, epigenetic instability at imprinted loci is not an unexpected finding in ESCs and has been reported by others (Humpherys et al., 2001, Pannetier and Feil, 2007, Stadtfeld et al., 2010). While it is of course possible that some have an additive effect and contribute to the severity of the phenotype, none of these other epigenetic changes correlate with the embryonic lethal phenotype as consistently as the changes in the *Dlk1-Dio3* imprinted locus. Therefore, at the very least, the reduced expression of *Gtl2, Rian, Mirg* and *Rtl1as* from the *Dlk1-Dio3* imprinted locus is a reliable marker for the compromised potential of mouse ESC clones.

There is evidence that the *Dlk1-Dio3* imprinted locus is important for embryonic development and that altered expression can change the functionality of both ESCs and iPSCs. Several knockout mouse models have shown that loss of expression of the maternally expressed genes results in pre- or postnatal lethality (Georgiades et al., 2000, Lin et al., 2007, Tevendale et al., 2006, Zhou et al., 2010). The *Dlk1-Dio3* imprinted locus has also been shown to be silenced in iPSCs that do not give rise to high percentage chimeras (Stadtfeld et al., 2010). Furthermore, similar to our results, it was described that ESCs with hypermethylation of the IG-DMR on the maternal allele have reduced developmental potential and embryos generated by tetraploid complementation with 4n blastocysts show developmental defects at e12.5-e15.5, including growth defects, brain malformation and muscle defects (Stelzer et al., 2016). We did not analyse muscle defects in the ESC(Comp) chimaeras but no obvious growth defects or brain malformations were observed. Functional annotation of the genes that are differentially expressed in our ESC(Comp) clones did, however, show that a large number of them are expressed in the brain (Supplementary data). We also cannot rule out a more minor phenotype in the brain. Interestingly, Stelzer et al. (Stelzer et al., 2016) did not report signs of haemorrhaging in their mutant embryos.

We did not find any relationship between the point in the sequential targeting pathway and the compromised potential of the clones. Indeed, the majority of the compromised clones were found very early in the gene targetting programme, and as non-compromised clones were identified and used successfully in subsequent gene targeting steps, the number of new compromised clones that we found actually fell. Thus, we think that the parental line used for the gene targeting probably contained ESCs with reduced expression of *Gtl2/Mirg/Rian* and some early targeted clones also had this problem but clones with normal expression levels of *Gtl2/Mirg/Rian* subsequently went on to be used in additional targeting steps with no problems.

We were able to rescue expression of *Gtl2*, *Rian* and *Mirg* by treating the ESC(Comp) clones with the DNMT inhibitor 5-azacytidine. To our knowledge, this is the first time that this has been demonstrated in ESCs. However, we did not succeed in fully rescuing the phenotype. Although a small number of chimaeric pups were born, their ESC contribution was very low. The reason for this remains to be determined, but we suspect that the 5-azacytidine may have had a detrimental effect on the ESCs such as global demethylation of other imprinted genes, which may have compromised their developmental potential. An alternative and potentially less damaging approach would be to treat the ESCs with ascorbic acid which has been reported to prevent *Gtl2* silencing during derivation of iPSCs (Stadtfeld et al., 2012) or to use the nuclease deficient dCas9 coupled with the TET catalytic domain (Liu et al., 2016, Xu et al., 2016). This system can specifically target the demethylating activity of the TET enzyme to the IG-DMR without affecting methylation at any other loci.

Little is known about the biological roles of the *Gtl2-Rian-Mirg* RNA species. In recent years they have been linked with several different biological processes, that could be involved in the lethality and haemorrhaging phenotype. One of those roles is inhibition of angiogenesis and tumour suppression. Many human tumours show hypermethylation at the IG-DMR of the *DLK1-DIO3* imprinted locus and consequently express very low levels of *MEG3* (the human homologue of mouse *Gtl2*), suggesting a role for *MEG3* in the onset and progression of cancer (Astuti et al., 2005, Braconi et al., 2011, Tian et al., 2015, Wang et al., 2017, Zhang et al., 2017, Zhang et al., 2003). Indeed, *MEG3* down-regulation or silencing has been associated with increased angiogenesis in human tumours (Zhang et al., 2017), *Gtl2* knockout mouse models (Gordon et al., 2010) and a rat model of tissue repair after ischaemic brain injury (Liu et al., 2017).This is regulated via activation of angiogenesis related genes including *Vegf*, *βFGF* and *TGF-β1*. Interestingly, a large number of genes that was differentially expressed between the ESC(Ctrl) and ESC(Comp) cells are associated with mammary tumours and some have also been shown to be involved in angiogenesis (Supplementary data). In addition, our ESC(Comp) chimaeras that do not express *Gtl2* and the other maternally expressed genes of the *Dlk1-Dio3* imprinted locus, present with haemorrhaging and a vascular phenotype which in part resembles some of the early steps of angiogenesis: dilated vessels and poor vascular integrity. It has also been shown that *Gtl2* suppresses vascular endothelial cell proliferation and impairs capillary formation (He et al., 2017). Hence, we hypothesise that loss of expression of *Gtl2*, *Rian, Mirg* and *Rtl1as* may cause dysregulated blood vessel development which subsequently results in haemorrhaging.

Other studies have shown an involvement of the maternally expressed genes of the *Dlk1-Dio3* imprinted locus in muscle development (Georgiades et al., 2000, Lin et al., 2007, Tevendale et al., 2006, Zhou et al., 2010) as well as in preserving long-term haematopoietic stem cell function by regulating mitochondrial biogenesis and metabolism (Qian et al., 2016).

Interestingly, one cellular pathway that is common to all those effects, is the PI3K/Akt/mTOR pathway. A connection between the *Dlk1-Dio3* imprinted locus and control of the PI3K/Akt/mTOR has specifically been demonstrated in angiogenesis (Qiu et al., 2016, Zhang et al., 2017) and mitochondrial biogenesis and metabolism (Qian et al., 2016). This link has not yet been shown in skeletal muscle development and it remains to be studied whether the maternally expressed genes of the *Dlk1-Dio3* imprinted locus always act through the PI3K/Akt/mTOR pathway or whether this is tissue specific. It is, however, known that the PI3K/Akt/mTOR pathway plays an important role in skeletal muscle development (Schiaffino et al., 2013) and it is possible that it is regulated, in part, through the *Dlk1-Dio3* imprinted locus. Given the central role of the PI3K/Akt/mTOR pathway throughout development and adult life, it is possible that further developmental processes are dysregulated in the ESC(Comp) chimaeras.

## 5 Conclusion and summary

The findings described in this paper will be very useful for scientists working in the field of ESC mediated transgenics. The availability of a large number of targeted ESCs from the Gene Targeting Consortium means that ESCs are still used for the generation of mouse models. Prior to using the ESCs for blastocyst injections, it is common practice to assess the quality of the cells by confirming the correct gene targeting event, analysing the karyotype and screening for pathogens. We suggest also including gene expression analysis of *Gtl2*, *Rian, Mirg* and *Rtl1as* in the pre-injection screening routine. Loss of imprinting is not an uncommon phenomenon in ESC culture and cells with aberrant silencing or downregulation of the maternally expressed genes of the *Dlk1-Dio3* imprinted locus will most certainly not be able to contribute to viable chimaeras. Elimination of compromised ESC clones before blastocyst injection will save resources and reduce unnecessary mouse use.

Furthermore, while this is still speculative and certainly needs to be investigated further, to our knowledge this is the first time that a potential link between loss of expression of the maternally expressed genes of the *Dlk1-Dio3* imprinted region and angiogenesis or vascular development in the mouse embryo has been shown.

## Declaration of Competing Interest

The authors declare that they have no known competing financial interests or personal relationships that could have appeared to influence the work reported in this paper.
