# Supplementary material for: Hypermethylation and reduced expression of Gtl2, Rian and Mirg at the Dlk1-Dio3 imprinted locus as a marker for poor developmental potential of mouse embryonic stem cells
Source: Stem Cell Res. 2020 Oct;48:101931. doi: 10.1016/j.scr.2020.101931 (PMC7567021; doi:10.1016/j.scr.2020.101931)
Supplement: Supplementary data 1 [file mmc1.docx]

**Supplementary Data**

Supplementary Table S1: qPCR reaction mix and thermal cycling conditions

| **Reaction mix (per reaction)** | **Step** | **Thermal cycling conditions** |
| --- | --- | --- |
| 7.5 ul SYBR Green master mix (PCR Biosystems)  0.15 ul 10 uM Forward Primer  0.15 ul 10uM Reverse Primer  6.2 ul DNA/RNA-free H_2_O  1 ul cDNA | DNA polymerase activation | 95°C for 2 min |
|  | 40 cycles of:  DNA denaturation  Primer annealing/extension | 40 cycles of:  95°C for 5 sec  60°C for 30 sec |
|  | Melt curve | 95°C for 15 sec  60°C for 1 min  95°C for 30 sec  60°C or 15 sec |

Supplementary Table S2: qPCR primer pairs

| **Gene** | **Forward primer (5’ – 3’)** | **Reverse primer (5’ – 3’)** |
| --- | --- | --- |
| *Hprt* | GATTAGCGATGATGAACCA | CCTCCCATCTCCTTCATGAC |
| *Gtl2* | AGTGCCTTGTAAATCGCCCG | CACCTACTGGGTGCTCACTG |
| *Rian* | TGTCACGGTCAGCTCTGTTC | ACCAAGGTGTACGCAACGAT |
| *Mirg* | TCGCTTACGACAACCGACAA | GGGTGAGAAGTTGGGGACTC |
| *Dlk1* | AGCACCTATGGGGCTGAATG | CACTTGTCACAGAGGGGACC |
| *Dio3* | TGCGTATCAGACGACAACCGTC | TGGAAGCCATCAGGTCGGACAA |
| *Rtl1/Rtl1as* | AGGCTATCAACGAAGGTCGC | TTCACCCGCAGCTCATCATT |
| *Rtl1/Rtl1as* (primer specific) | GAGAGTGGACCCCTACCACA | GGCAAACCTCTCATCCATGT |

Supplementary Table S3: List of differentially expressed genes between ESC(Ctrl) and ESC(Comp) clones according to FDR.

| Gene identifier | Gene ID | The logarithm (to base 2) of the fold change | p value adjusted for multiple testing with the Benjamini-Hochberg procedure which controls false discovery rate (FDR) |
| --- | --- | --- | --- |
| ENSMUSG00000097391 | *Mirg* | -3.28 | 3.39036E-118 |
| ENSMUSG00000097451 | *Rian* | -3.12 | 1.41907E-105 |
| ENSMUSG00000021268 | *Meg3* | -2.32 | 3.70532E-56 |
| ENSMUSG00000097961 | *Gm27000* | -1.91 | 1.54408E-37 |
| ENSMUSG00000085925 | *Rtl1* | -1.65 | 3.38426E-28 |
| ENSMUSG00000035161 | *Ints6* | -0.51 | 4.04129E-12 |
| ENSMUSG00000066441 | *Rdh11* | 0.34 | 9.92431E-11 |
| ENSMUSG00000026398 | *Nr5a2* | -0.64 | 2.29092E-10 |
| ENSMUSG00000030693 | *Klk10* | -0.99 | 5.79963E-10 |
| ENSMUSG00000027313 | *Chac1* | 0.99 | 6.40701E-10 |
| ENSMUSG00000038914 | *Dido1* | -0.47 | 9.58670E-10 |
| ENSMUSG00000006651 | *Aplp1* | 0.42 | 1.32392E-08 |
| ENSMUSG00000022126 | *Acod1* | -0.86 | 4.06019E-08 |
| ENSMUSG00000003378 | *Grik5* | 0.76 | 7.07590E-08 |
| ENSMUSG00000031530 | *Dusp4* | 0.57 | 2.34888E-07 |
| ENSMUSG00000051855 | *Mest* | -0.41 | 4.07583E-07 |
| ENSMUSG00000059901 | *Adamts14* | 0.65 | 4.57407E-07 |
| ENSMUSG00000042408 | *Zmym6* | 0.70 | 7.86636E-07 |
| ENSMUSG00000007038 | *Neu1* | 0.57 | 1.41726E-06 |
| ENSMUSG00000021823 | *Vcl* | -0.41 | 1.41726E-06 |
| ENSMUSG00000034245 | *Hdac11* | 0.67 | 1.64122E-06 |
| ENSMUSG00000031425 | *Plp1* | 0.79 | 1.93576E-06 |
| ENSMUSG00000022750 | *Klhl22* | 0.43 | 3.06328E-06 |
| ENSMUSG00000010064 | *Slc38a3* | 0.67 | 3.09567E-06 |
| ENSMUSG00000022718 | *Dgcr8* | -0.44 | 4.20888E-06 |
| ENSMUSG00000038331 | *Satb2* | -0.67 | 4.48907E-06 |
| ENSMUSG00000066595 | *Flvcr1* | -0.40 | 7.24940E-06 |
| ENSMUSG00000027498 | *Cstf1* | -0.20 | 9.25323E-06 |
| ENSMUSG00000053062 | *Jam2* | -0.30 | 1.27623E-05 |
| ENSMUSG00000027397 | *Slc20a1* | 0.28 | 1.31079E-05 |
| ENSMUSG00000002015 | *Bcap31* | -0.23 | 1.54399E-05 |
| ENSMUSG00000026955 | *Sapcd2* | 0.55 | 1.54399E-05 |
| ENSMUSG00000058318 | *Phf21a* | 0.44 | 1.54399E-05 |
| ENSMUSG00000039804 | *Ncoa5* | -0.25 | 1.64364E-05 |
| ENSMUSG00000018340 | *Anxa6* | -0.25 | 1.75965E-05 |
| ENSMUSG00000018983 | *E2f2* | -0.53 | 1.75965E-05 |
| ENSMUSG00000022453 | *Naga* | 0.46 | 1.75965E-05 |
| ENSMUSG00000036568 | *Bicral* | -0.22 | 1.92311E-05 |
| ENSMUSG00000047539 | *Fbxo28* | -0.29 | 2.16441E-05 |
| ENSMUSG00000030103 | *Bhlhe40* | 0.49 | 2.39771E-05 |
| ENSMUSG00000031383 | *Dusp9* | 0.37 | 2.46798E-05 |
| ENSMUSG00000058126 | *Tpm3-rs7* | -0.19 | 2.86325E-05 |
| ENSMUSG00000023079 | *Gtf2ird1* | 0.30 | 2.95537E-05 |
| ENSMUSG00000001521 | *Tulp3* | 0.33 | 3.05609E-05 |
| ENSMUSG00000081683 | *Fzd10* | -0.62 | 3.05609E-05 |
| ENSMUSG00000020326 | *Ccng1* | -0.49 | 3.06347E-05 |
| ENSMUSG00000026339 | *Ccdc93* | 0.37 | 3.11378E-05 |
| ENSMUSG00000038679 | *Trps1* | -0.57 | 3.11378E-05 |
| ENSMUSG00000029071 | *Dvl1* | 0.43 | 3.18339E-05 |
| ENSMUSG00000030102 | *Itpr1* | 0.40 | 3.24629E-05 |
| ENSMUSG00000029090 | *Adgra3* | 0.24 | 3.31345E-05 |
| ENSMUSG00000035498 | *Cdcp1* | 0.53 | 3.51987E-05 |
| ENSMUSG00000058230 | *Arhgap35* | -0.35 | 3.51987E-05 |
| ENSMUSG00000034486 | *Gbx2* | -0.69 | 3.60189E-05 |
| ENSMUSG00000006649 | *Nphs1* | 0.62 | 3.66076E-05 |
| ENSMUSG00000028957 | *Per3* | 0.61 | 3.66076E-05 |
| ENSMUSG00000027999 | *Pla2g12a* | 0.39 | 3.72637E-05 |
| ENSMUSG00000042406 | *Atf4* | 0.28 | 4.61101E-05 |
| ENSMUSG00000028613 | *Lrp8* | 0.53 | 4.84392E-05 |
| ENSMUSG00000025930 | *Msc* | -0.50 | 4.97405E-05 |
| ENSMUSG00000034263 | *Ints14* | -0.29 | 4.97405E-05 |
| ENSMUSG00000026080 | *Chst10* | 0.60 | 5.15732E-05 |
| ENSMUSG00000036915 | *Kirrel2* | 0.63 | 5.19281E-05 |
| ENSMUSG00000026374 | *Tsn* | -0.19 | 5.28209E-05 |
| ENSMUSG00000027820 | *Mme* | -0.46 | 5.28209E-05 |
| ENSMUSG00000040276 | *Pacsin1* | 0.46 | 5.28209E-05 |
| ENSMUSG00000029636 | *Wasf3* | 0.31 | 5.42402E-05 |
| ENSMUSG00000062170 | *Fmr1nb* | -0.70 | 5.42402E-05 |
| ENSMUSG00000045679 | *Pqlc3* | -0.49 | 5.75037E-05 |
| ENSMUSG00000057193 | *Slc44a2* | 0.22 | 6.64279E-05 |
| ENSMUSG00000030800 | *Prss8* | 0.44 | 7.09909E-05 |
| ENSMUSG00000028337 | *Coro2a* | 0.48 | 8.10998E-05 |
| ENSMUSG00000096740 | *Lbhd1* | -0.36 | 8.10998E-05 |
| ENSMUSG00000002324 | *Rec8* | 0.44 | 8.34708E-05 |
| ENSMUSG00000032470 | *Mras* | -0.53 | 8.34708E-05 |
| ENSMUSG00000042724 | *Map3k9* | 0.44 | 8.34708E-05 |
| ENSMUSG00000032633 | *Flcn* | 0.28 | 8.38959E-05 |
| ENSMUSG00000008496 | *Pou2f2* | 0.64 | 8.41465E-05 |
| ENSMUSG00000041025 | *Iffo2* | 0.38 | 8.41465E-05 |
| ENSMUSG00000005102 | *Eif2ak4* | 0.24 | 8.65905E-05 |
| ENSMUSG00000032092 | *Mpzl2* | -0.38 | 8.65905E-05 |
| ENSMUSG00000021891 | *Mettl6* | -0.36 | 9.08074E-05 |
| ENSMUSG00000028184 | *Adgrl2* | 0.26 | 9.65396E-05 |
| ENSMUSG00000051257 | *Trap1a* | -0.28 | 0.00010 |
| ENSMUSG00000025795 | *Rassf3* | 0.30 | 0.00010 |
| ENSMUSG00000050335 | *Lgals3* | -0.37 | 0.00010 |
| ENSMUSG00000030609 | *Aen* | -0.28 | 0.00010 |
| ENSMUSG00000020088 | *Sar1a* | -0.20 | 0.00011 |
| ENSMUSG00000037129 | *Tmprss13* | 0.70 | 0.00011 |
| ENSMUSG00000025236 | *Adpgk* | 0.27 | 0.00011 |
| ENSMUSG00000006056 | *Calcoco2* | -0.58 | 0.00011 |
| ENSMUSG00000021905 | *Dph3* | -0.24 | 0.00011 |
| ENSMUSG00000027331 | *Knstrn* | 0.31 | 0.00011 |
| ENSMUSG00000005503 | *Evx1* | -0.70 | 0.00012 |
| ENSMUSG00000075595 | *Zfp652* | 0.34 | 0.00013 |
| ENSMUSG00000004631 | *Sgce* | 0.69 | 0.00013 |
| ENSMUSG00000006818 | *Sod2* | -0.27 | 0.00013 |
| ENSMUSG00000020674 | *Pxdn* | 0.38 | 0.00013 |
| ENSMUSG00000020808 | *Pimreg* | 0.38 | 0.00014 |
| ENSMUSG00000030653 | *Gm45837* | 0.53 | 0.00014 |
| ENSMUSG00000017057 | *Il13ra1* | -0.46 | 0.00014 |
| ENSMUSG00000029571 | *Tmem106b* | 0.41 | 0.00014 |
| ENSMUSG00000034248 | *Slc25a37* | 0.44 | 0.00015 |
| ENSMUSG00000079487 | *Med12* | 0.32 | 0.00015 |
| ENSMUSG00000030970 | *Ctbp2* | 0.16 | 0.00015 |
| ENSMUSG00000044934 | *Zfp367* | 0.54 | 0.00015 |
| ENSMUSG00000033105 | *Lss* | 0.21 | 0.00015 |
| ENSMUSG00000000532 | *Acvr1b* | 0.36 | 0.00015 |
| ENSMUSG00000007379 | *Dennd2c* | -0.29 | 0.00015 |
| ENSMUSG00000033998 | *Kcnk1* | 0.38 | 0.00015 |
| ENSMUSG00000036667 | *Tcaf1* | 0.50 | 0.00015 |
| ENSMUSG00000038696 | *Mapkap1* | 0.27 | 0.00015 |
| ENSMUSG00000039682 | *Lap3* | -0.15 | 0.00015 |
| ENSMUSG00000066878 | *Gm10184* | -0.38 | 0.00015 |
| ENSMUSG00000020883 | *Fbxl20* | 0.42 | 0.00015 |
| ENSMUSG00000036026 | *Tmem63b* | 0.30 | 0.00015 |
| ENSMUSG00000042066 | *Tmcc2* | 0.58 | 0.00016 |
| ENSMUSG00000048922 | *Cdca2* | -0.25 | 0.00016 |
| ENSMUSG00000023809 | *Rps6ka2* | 0.44 | 0.00017 |
| ENSMUSG00000037706 | *Cd81* | -0.30 | 0.00017 |
| ENSMUSG00000021403 | *Serpinb9b* | -0.61 | 0.00017 |
| ENSMUSG00000058761 | *Rnf169* | -0.58 | 0.00017 |
| ENSMUSG00000037279 | *Ovol2* | -0.59 | 0.00017 |
| ENSMUSG00000071645 | *Tut1* | -0.32 | 0.00018 |
| ENSMUSG00000041119 | *Pde9a* | 0.49 | 0.00018 |
| ENSMUSG00000040435 | *Ppp1r15a* | 0.31 | 0.00018 |
| ENSMUSG00000068154 | *Insm1* | 0.66 | 0.00018 |
| ENSMUSG00000024427 | *Spry4* | -0.51 | 0.00020 |
| ENSMUSG00000034917 | *Tjp3* | 0.50 | 0.00020 |
| ENSMUSG00000038893 | *Fam117a* | 0.51 | 0.00020 |
| ENSMUSG00000021518 | *Ptdss1* | -0.26 | 0.00021 |
| ENSMUSG00000028745 | *Capzb* | -0.16 | 0.00022 |
| ENSMUSG00000028082 | *Sh3d19* | 0.30 | 0.00023 |
| ENSMUSG00000030177 | *Ccdc77* | 0.31 | 0.00023 |
| ENSMUSG00000022206 | *Npr3* | -0.60 | 0.00023 |
| ENSMUSG00000052056 | *Zfp217* | -0.36 | 0.00023 |
| ENSMUSG00000032531 | *Amotl2* | 0.46 | 0.00025 |
| ENSMUSG00000038059 | *Smim3* | -0.61 | 0.00026 |
| ENSMUSG00000044250 | *Pced1b* | -0.32 | 0.00026 |
| ENSMUSG00000022679 | *Mpv17l* | 0.44 | 0.00027 |
| ENSMUSG00000024268 | *Celf4* | 0.36 | 0.00027 |
| ENSMUSG00000001300 | *Efnb2* | 0.50 | 0.00027 |
| ENSMUSG00000032254 | *Kif23* | -0.31 | 0.00027 |
| ENSMUSG00000044037 | *Als2cl* | 0.40 | 0.00027 |
| ENSMUSG00000053898 | *Ech1* | -0.22 | 0.00027 |
| ENSMUSG00000026817 | *Ak1* | -0.34 | 0.00027 |
| ENSMUSG00000027341 | *Tmem230* | 0.37 | 0.00027 |
| ENSMUSG00000056531 | *Ccdc18* | 0.55 | 0.00028 |
| ENSMUSG00000024096 | *Ralbp1* | -0.29 | 0.00029 |
| ENSMUSG00000032178 | *Ilf3* | 0.17 | 0.00030 |
| ENSMUSG00000028977 | *Casz1* | 0.49 | 0.00031 |
| ENSMUSG00000027238 | *Frmd5* | 0.49 | 0.00031 |
| ENSMUSG00000029922 | *Mkrn1* | 0.17 | 0.00031 |
| ENSMUSG00000020953 | *Coch* | -0.62 | 0.00033 |
| ENSMUSG00000004113 | *Cacna1b* | 0.59 | 0.00033 |
| ENSMUSG00000032515 | *Csrnp1* | 0.37 | 0.00033 |
| ENSMUSG00000019998 | *Stx7* | 0.40 | 0.00033 |
| ENSMUSG00000002102 | *Psmc3* | -0.14 | 0.00035 |
| ENSMUSG00000029311 | *Hsd17b11* | -0.37 | 0.00035 |
| ENSMUSG00000048458 | *Fam212b* | -0.64 | 0.00035 |
| ENSMUSG00000078779 | *Zfp59* | -0.48 | 0.00035 |
| ENSMUSG00000046718 | *Bst2* | 0.34 | 0.00035 |
| ENSMUSG00000092035 | *Peg10* | 0.64 | 0.00037 |
| ENSMUSG00000022808 | *Snx4* | -0.21 | 0.00038 |
| ENSMUSG00000024146 | *Cript* | -0.23 | 0.00038 |
| ENSMUSG00000034435 | *Tmem30b* | 0.42 | 0.00040 |
| ENSMUSG00000027496 | *Aurka* | -0.27 | 0.00041 |
| ENSMUSG00000036825 | *Ssx2ip* | 0.36 | 0.00041 |
| ENSMUSG00000047466 | *8030462N17Rik* | -0.25 | 0.00041 |
| ENSMUSG00000020823 | *Sec14l1* | 0.21 | 0.00042 |
| ENSMUSG00000061455 | *Stx17* | 0.43 | 0.00042 |
| ENSMUSG00000018678 | *Sp2* | -0.30 | 0.00044 |
| ENSMUSG00000032897 | *Nfyc* | -0.26 | 0.00044 |
| ENSMUSG00000053846 | *Lipg* | 0.64 | 0.00044 |
| ENSMUSG00000036617 | *Etl4* | 0.39 | 0.00044 |
| ENSMUSG00000060985 | *Tdrd5* | 0.47 | 0.00045 |
| ENSMUSG00000042272 | *Sestd1* | 0.33 | 0.00047 |
| ENSMUSG00000036989 | *Trim3* | 0.45 | 0.00047 |
| ENSMUSG00000069769 | *Msi2* | 0.27 | 0.00047 |
| ENSMUSG00000029055 | *Plch2* | 0.59 | 0.00047 |
| ENSMUSG00000029250 | *Polr2b* | -0.18 | 0.00047 |
| ENSMUSG00000020580 | *Rock2* | -0.52 | 0.00047 |
| ENSMUSG00000019997 | *Ctgf* | 0.33 | 0.00049 |
| ENSMUSG00000029580 | *Actb* | -0.28 | 0.00049 |
| ENSMUSG00000031403 | *Dkc1* | -0.20 | 0.00049 |
| ENSMUSG00000037465 | *Klf10* | 0.35 | 0.00049 |
| ENSMUSG00000029076 | *Sdf4* | -0.16 | 0.00049 |
| ENSMUSG00000031770 | *Herpud1* | 0.56 | 0.00050 |
| ENSMUSG00000045136 | *Tubb2b* | -0.23 | 0.00050 |
| ENSMUSG00000024247 | *Pkdcc* | 0.63 | 0.00050 |
| ENSMUSG00000062761 | *Zfp512* | 0.31 | 0.00050 |
| ENSMUSG00000068270 | *Shroom4* | 0.44 | 0.00050 |
| ENSMUSG00000050931 | *Sgms2* | -0.57 | 0.00051 |
| ENSMUSG00000041649 | *Klf8* | -0.54 | 0.00052 |
| ENSMUSG00000026479 | *Lamc2* | -0.50 | 0.00052 |
| ENSMUSG00000062270 | *Morf4l1* | -0.23 | 0.00052 |
| ENSMUSG00000057110 | *Cntrl* | 0.33 | 0.00053 |
| ENSMUSG00000001467 | *Cyp51* | 0.22 | 0.00054 |
| ENSMUSG00000021559 | *Dapk1* | 0.37 | 0.00054 |
| ENSMUSG00000025287 | *Acot9* | -0.24 | 0.00054 |
| ENSMUSG00000028932 | *Psmc2* | -0.16 | 0.00054 |
| ENSMUSG00000029859 | *Epha1* | 0.42 | 0.00054 |
| ENSMUSG00000091421 | *Gm4202* | -0.23 | 0.00055 |
| ENSMUSG00000048833 | *Slc39a9* | -0.27 | 0.00059 |
| ENSMUSG00000061731 | *Ext1* | -0.26 | 0.00059 |
| ENSMUSG00000047412 | *Zbtb44* | -0.25 | 0.00062 |
| ENSMUSG00000072949 | *Acot1* | 0.49 | 0.00062 |
| ENSMUSG00000055897 | *Ppp4r1l-ps* | 0.49 | 0.00064 |
| ENSMUSG00000070639 | *Lrrc8b* | 0.43 | 0.00064 |
| ENSMUSG00000034601 | *2700049A03Rik* | 0.33 | 0.00067 |
| ENSMUSG00000021733 | *Slc4a7* | 0.31 | 0.00067 |
| ENSMUSG00000025427 | *Rnf165* | 0.39 | 0.00067 |
| ENSMUSG00000001248 | *Gramd1a* | 0.31 | 0.00068 |
| ENSMUSG00000039328 | *Rnf122* | 0.44 | 0.00068 |
| ENSMUSG00000041935 | *AW549877* | 0.38 | 0.00070 |
| ENSMUSG00000034391 | *Fbxo15* | -0.33 | 0.00070 |
| ENSMUSG00000041975 | *Mettl8* | 0.40 | 0.00071 |
| ENSMUSG00000023960 | *Enpp5* | 0.47 | 0.00071 |
| ENSMUSG00000056201 | *Cfl1* | -0.18 | 0.00071 |
| ENSMUSG00000040918 | *Slc19a2* | -0.42 | 0.00071 |
| ENSMUSG00000029461 | *Fam168a* | -0.41 | 0.00075 |
| ENSMUSG00000003341 | *Atp8b3* | 0.61 | 0.00076 |
| ENSMUSG00000039686 | *Zer1* | 0.32 | 0.00076 |
| ENSMUSG00000028607 | *Cpt2* | 0.40 | 0.00082 |
| ENSMUSG00000020515 | *Cnot8* | 0.27 | 0.00085 |
| ENSMUSG00000027889 | *Ampd2* | 0.20 | 0.00086 |
| ENSMUSG00000040842 | *Szrd1* | -0.20 | 0.00086 |
| ENSMUSG00000022286 | *Grhl2* | 0.33 | 0.00086 |
| ENSMUSG00000004567 | *Mcoln1* | 0.55 | 0.00087 |
| ENSMUSG00000024190 | *Dusp1* | 0.30 | 0.00087 |
| ENSMUSG00000041890 | *Git2* | 0.31 | 0.00087 |
| ENSMUSG00000060147 | *Serpinb6a* | -0.21 | 0.00087 |
| ENSMUSG00000042216 | *Sgsm1* | 0.46 | 0.00088 |
| ENSMUSG00000038884 | *A230050P20Rik* | 0.39 | 0.00089 |
| ENSMUSG00000042988 | *Notum* | -0.37 | 0.00089 |
| ENSMUSG00000037211 | *Spry1* | 0.43 | 0.00091 |
| ENSMUSG00000020114 | *Cand1* | -0.23 | 0.00091 |
| ENSMUSG00000042350 | *Arel1* | 0.28 | 0.00091 |
| ENSMUSG00000030231 | *Plekha5* | 0.39 | 0.00093 |
| ENSMUSG00000022218 | *Tgm1* | -0.48 | 0.00093 |
| ENSMUSG00000029084 | *Cd38* | -0.51 | 0.00093 |
| ENSMUSG00000050628 | *Ubald2* | 0.35 | 0.00095 |
| ENSMUSG00000022610 | *Mapk12* | 0.51 | 0.00095 |
| ENSMUSG00000040025 | *Ythdf2* | -0.18 | 0.00095 |
| ENSMUSG00000027651 | *Rprd1b* | -0.23 | 0.00096 |
| ENSMUSG00000033632 | *AW554918* | 0.50 | 0.00096 |
| ENSMUSG00000097908 | *4933404O12Rik* | 0.26 | 0.00099 |
| ENSMUSG00000017376 | *Nlk* | 0.32 | 0.00099 |
| ENSMUSG00000026753 | *Ppp6c* | -0.20 | 0.00099 |
| ENSMUSG00000064194 | *Zfp936* | -0.34 | 0.00099 |
| ENSMUSG00000099875 | *Rbm3-ps* | -0.31 | 0.00099 |


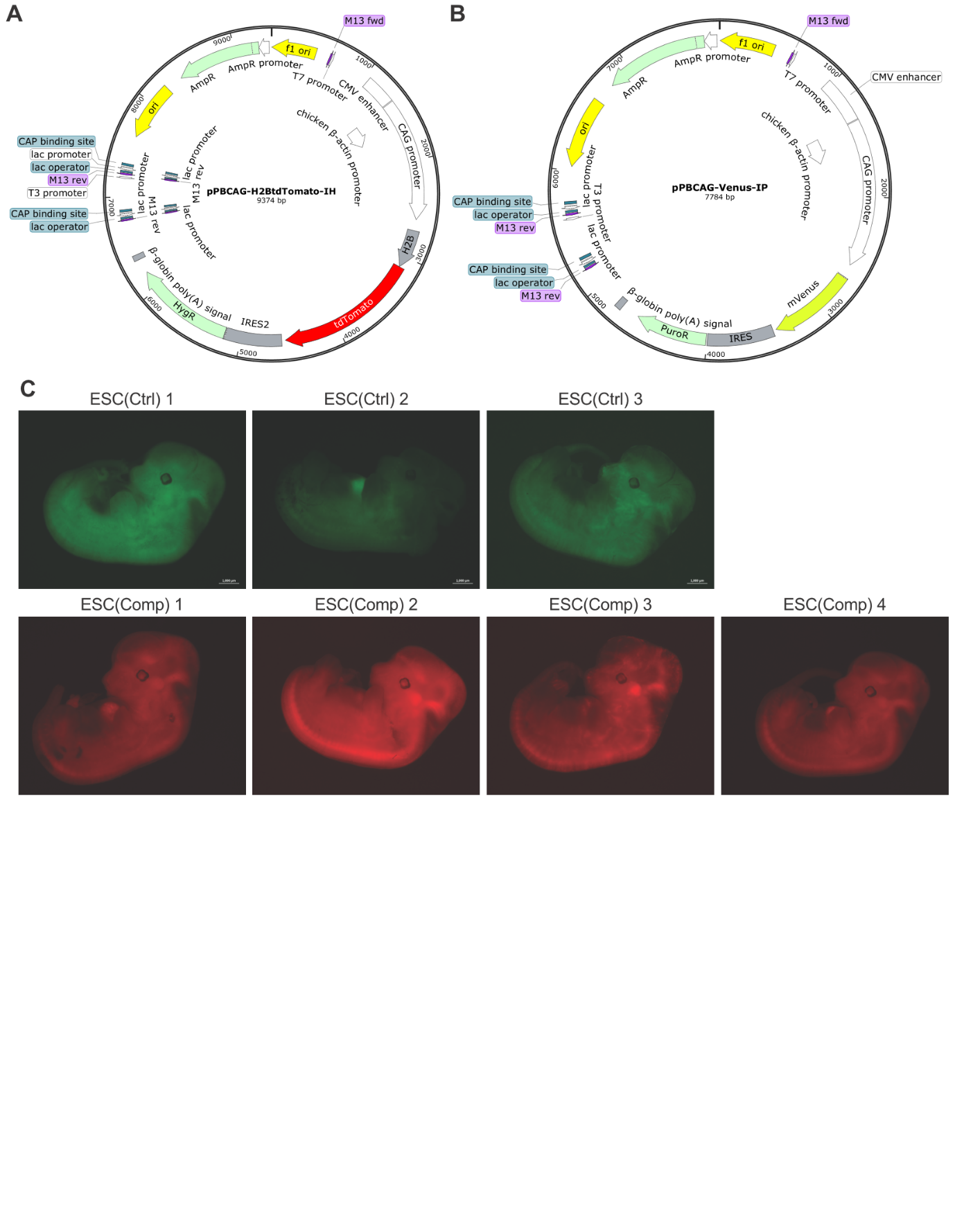


**Supplementary Figure S1: Cell labelling using the Piggy Bac system.**

1. pPBCAG-H2BtdTomato-IH vector containing the tdTomato transgene, as well as the hygromycin resistance gene. (B) pPBCAG-Venus-IP vector containing the Venus transgene as well as the puromycin resistance gene. (C) Images of e13.5 chimaeras produced with the fluorescently labeled cell clones.

**Functional annotation of RNA sequencing results**

We also analysed overrepresented Gene Ontology (GO) terms related to biological processes and KEGG pathways of the differentially expressed genes, as well as enriched UniProt tissue terms using the DAVID Functional Annotation Tool. The most significant GO terms and KEGG pathways are presented in Supplementary Tables S4 and S5. UniProt tissue terms that were overrepresented among the differentially expressed genes are presented in Supplementary Table S6. While none of these annotations are significantly associated (p <0.05; adjusted for multiple testing with the Benjamini-Hochberg procedure) with the list of genes that are up- or downregulated in the ESC(Comp) samples, some processes that they are involved in as shown by functional analysis include regulation of transcription and MAPK signaling (Supplementary Tables S4 and S5). Further, it should be noted that a large number of these genes are typically expressed in the brain and in mammary tumours (Supplementary Table S6). We also identified eight differentially expressed genes that have been shown to be involved in angiogenesis and vascularization including *Efnb2* (Wang et al., 2010)*, Epha1* (Mosch et al., 2010)*, Ctgf* (Pi et al., 2012)*, Rock2* (Bryan et al., 2010) and *Ovol2* (Unezaki et al., 2007).

**References.**

Bryan, B.A., Dennstedt, E., Mitchell, D.C., Walshe, T.E., Noma, K., Loureiro, R., Saint-Geniez, M., Campaigniac, J.-P., Liao, J.K., D’Amore, P.A., 2010. RhoA/ROCK signaling is essential for multiple aspects of VEGF-mediated angiogenesis. FASEB J. 24, 3186–3195. <https://doi.org/10.1096/fj.09-145102>.

Mosch, B., Reissenweber, B., Neuber, C., Pietzsch, J., 2010. *Eph* receptors and ephrin ligands: important players in angiogenesis and tumor angiogenesis. J. Oncol. 2010, 135285. <https://doi.org/10.1155/2010/135285>.

Pi, L., Shenoy, A.K., Liu, J., Kim, S., Nelson, N., Xia, H., Hauswirth, W.W., Petersen, B.E., Schultz, G.S., Scott, E.W., 2012. CCN2/CTGF regulates neovessel formation via targeting structurally conserved cystine knot motifs in multiple angiogenic regulators. FASEB J. 26, 3365–3379. <https://doi.org/10.1096/fj.11-200154>.

Unezaki, S., Horai, R., Sudo, K., Iwakura, Y., Ito, S., 2007. *Ovol2/Movo*, a homologue of Drosophila ovo, is required for angiogenesis, heart formation and placental development in mice. Genes Cells 12, 773–785. <https://doi.org/10.1111/j.1365-2443.2007.01084.x>

Wang, Y., Nakayama, M., Pitulescu, M.E., Schmidt, T.S., Bochenek, M.L., Sakakibara, A., Adams, S., Davy, A., Deutsch, U., Lüthi, U., Barberis, A., Benjamin, L.E., Mäkinen, T., Nobes, C.D., Adams, R.H., 2010. Ephrin-B2 controls VEGF-induced angiogenesis and lymphangiogenesis. Nature 465, 483–486. https://doi.org/10.1038/nature09002

Supplementary Table S4: GO Terms Biological Processes of up- and downregulated genes in ESC(Comp) clones

| Term / description | Count | Genes | P-value  (Benjamini Hochberg) |
| --- | --- | --- | --- |
| **Upregulated in ESC(Comp)** | | | |
| GO:0000188  Inactivation of MAPK activity | 3 | *Dusp1, Dusp9, Dusp4* | 0.9703 |
| GO:1900006  Positive regulation of dendrite development | 3 | *Lrp8, Tmem106b, Pacsin1* | 0.9401 |
| GO:0046777  Protein autophosphorylation | 6 | *Acvr1b, Eif2ak4, Nlk, Dapk1, Epha1, Map3k9* | 0.8957 |
| GO:0006417  Regulation of translation | 5 | *Eif2ak4, Aplp1, Cnot8, Dapk1, Ppp1r15a* | 0.8486 |
| GO:0070373  Negative regulation of ERK1 and ERK2 cascade | 4 | *Dusp1, Dusp4, Flcn, Spry1* | 0.8033 |
| GO:0006810  Transport | 22 | *Atp8b3, Grik5, Cacna1b, Mcoln1, Slc38a3, Sec14l1, Slc4a7, Pkdcc, Ccdc93, Slc20a1, Cpt2, Tmem106b, Itpr1, Kcnk1, Slc25a37, Tmem30b, Tmem63b, Tcaf1, Trim3, Slc44a2, Stx17, Lrrc8b* | 0.8318 |
| GO:0014003  Oligodendrocyte development | 3 | *Wasf3, Hdac11, Med12* | 0.7978 |
| GO:0034976  Response to endoplasmic reticulum stress | 4 | *Eif2ak4, Herpud1, Ppp1r15a, Atf4* | 0.7744 |
| GO:0006468  Protein phosphorylation | 10 | *Acvr1b, Eif2ak4, Nlk, Dapk1, Mapk12, Rps6ka2, Pkdcc, Epha1, Ilf3, Map3k9* | 0.7996 |
| **Downregulated in ESC(Comp)** | | | |
| GO:0045899  Positive regulation of RNA polymerase II transcriptional preinitiation complex assembly | 3 | *Psmc3, Cand1, Psmc2* | 0.5837 |
| GO:0006351  Transcription, DNA-templated | 17 | *Sp2, E2f2, Nr5a2, Rprd1b, Polr2, Nfyc, Gbx2, Ovol2, Satb2, Trps1, Dido1, Ncoa5, Klf8, btb44, Arhgap35, Morf4l1* | 0.9975 |
| GO:0001755  Neural crest cell migration | 3 | *Gbx2, Ovol2, Cfl1* | 0.9907 |
| GO:0001842  Neural fold formation | 2 | *Ovol2, Cfl1* | 0.9706 |
| GO:0032956  Regulation of actin cytoskeleton organization | 3 | *Rock2, Arhgap35, Fzd10* | 0.9459 |
| GO:0006355  Regulation of transcription, DNA-templated | 19 | *Evx1, Sp2, E2f2, Msc, Nr5a2, Rprd1b, Nfyc, Gbx2, Ovol2, Satb2, Trps1, Ncoa5, Klf8, Zbtb44, Zfp217, Arhgap35, Morf4l1, Zfp936, Zfp59* | 0.9304 |
| GO:0007411  Axon guidance | 4 | *Meg3, Gbx2, Arhgap35, Ext1* | 0.9550 |
| GO:0019538  Protein metabolic process | 2 | *Tgm1, Lap3* | 0.9855 |
| GO:0016180  snRNA processing | 2 | *Ints6, Tut1* | 0.9830 |

Supplementary Table S5: KEGG pathways of up- and downregulated genes in ESC(Comp) clones

| Term / description | Count | Genes | P-value  (Benjamini Hochberg) |
| --- | --- | --- | --- |
| **Upregulated ESC(Comp)** | | | |
| mmu04010  MAPK signaling pathway | 8 | *Cacna1b, Nlk, Mapk12, Prs6ka2, Dusp1, Dusp9, Dusp4, Atf4* | 0.1813 |
| mmu04728  Dopaminergic synapse | 4 | *Cacna1b, Mapk12, Itpr1, Atf4* | 0.9864 |
| mmu04550  Signaling pathways regulating pluripotency of stem cells | 4 | *Acvr1b, Mapk12, Dvl1, Dusp9* | 0.9542 |
| mmu04720  Long-term potentiation | 3 | *Rps6ka2, Itpr1, Atf4* | 0.9207 |
| mmu04520  Adherens junction | 3 | *Nlk, Wasf3, Ssx2ip* | 0.9065 |
| **Downregulated ESC(Comp)** | | | |
| mmu04670  Leukocyte transendothelial migration | 5 | *Rock2, Vcl, Actb, Jam2, Arhgap35* | 0.2295 |
| mmu04810  Regulation of actin cytoskeleton | 6 | *Rock2, Vcl, Actb, Mras, Cfl1, Arhgap35* | 0.1726 |
| mmu04510  Focal adhesion | 5 | *Rock2, Vcl, Lamc2, Actb, Arhgap35* | 0.4596 |
| mmu05146  Amoebiasis | 4 | *Serpinb9b, Vcl, Lamc2, Serpinb6a* | 0.3839 |
| mmu05205  Proteoglycans in cancer | 4 | *Rock2, Actb, Mras, Fzd10* | 0.7926 |

Supplementary Table S6: Tissue expression of genes that are up- and downregulated in ESC(Comp) clones

| Tissue | Count | Genes | P-value  (Benjamini Hochberg) |
| --- | --- | --- | --- |
| **Upregulated ESC(Comp)** | | | |
| Mammary tumor | 34 | *Gramd1a, Mcoln1, Fam64a, Grhl2, Naga, Klhl22, Adpgk, Rassf3, Chst10, Chac1, Knstrn, Tmem230, Slc20a1, Ampd2, Pde2a, Ilf3, Amotl2, Lss, Cdcp1, Tmem63b, Tcaf1, Tmprss13, Mapkap1, A230050P20Rik, Rnf122, Iffo2, AW549877, Mettl8, Sestd1, Bst2, Cntrl, Phf21a, Zfp512, Rhd11* | 0.1460 |
| Retina | 11 | *Aplp1, Slc38a3, Rps6ka2, Frmd5, Adgrl2, Plch2, Wasf3, Itpr1, Plp1, Tcaf1, Trim3* | 0.4237 |
| Brain cortex | 10 | *Cacna1b, Mcoln1, Nlk, Stx7, Mpv17l, Celf4, Lss, Kcnk1, Ssx2ip, Tmcc2* | 0.4880 |
| Brain | 66 | *Acvr1b, Gramd1a, Efnb2, Tulp3, Grik5, Cacna1b, Mcoln1, Eif2ak4, Nphs1, Aplp1, Slc38a3, Nlk, Stx7, Pxdn, Dapk1, Slc4a7, Gtf2ird1, Rps6ka2, Enpp5, Pkdcc, Celf4, Chst10, Frmd5, Tmem230, Slc20a1, Ampd2, Adgrl2, Coro2a, Cpt2, Lrp8, Per3, Plch2, Dvl1, Adgra3, Itpr1, Plekha5, Pde2a, Plp1, AW554918, Kcnk1, Tmem30b, 2700049A03Rik, Etl4, Kirrel2, Trim3, Mapkap1, Fam117a, Pacsin1, Ppp1r15a, Pde9a, Git2, Tmcc2, Sgsm1, Arel1, Atf4, Zmym6, Als2cl, Ubald2, Cntrl, Phf21a, Adamts14, Msi2, Lrrc8b, Zfp652, Med12, Sgce* | 0.5408 |
| Embryonic tail | 7 | *Eif2ak4, Adgrl2, Dvl1, AW554918, Etl4, Ssx2ip, Phf21a* | 0.5964 |
| Olfactory epithelium | 7 | *Grhl2, Pla2g12a, Adgra3, Tmem106b, Kirrel2, Mapkap1, Zer1* | 0.6907 |
| Eye | 17 | *Celf4, Ccdc93, Sapcd2, Plch2, Wasf3, Ctbp2, Dusp4, Tcaf1, Mapkap1, Pacsin1, Sgsm1, Arel1, Atf4, Zmym6, Map3k9, Zfp512, Insm1* | 0.7644 |
| Placenta | 10 | *Acvr1b, Cyp51, Mcoln1, Dapk1, Dusp1, Adpgk, Dusp9, Mapkap1, Ppp1r15a, Sgsm1* | 0.7292 |
| **Downregulated ESC(Comp)** | | | |
| Amnion | 7 | *Psmc3, Anxa6, Psmc2, Lap3, Trap1a, Mest, Serpinb6a* | 0.6971 |
| Bone marrow | 16 | *Bcap31, Psmc3, Sod2, Anxa6, Ccng1, Tsn, Psmc2, Sdf4, Cd38, Actb, Lap3, Ythdf2, Pqlc3, Slc39a9, Lgals3, Morf4l1* | 0.7661 |
| Activated spleen | 8 | *Sp2, E2f2, Coch, Serpinb9b, Capzb, Nfyc, Smim3, Zfp217* | 0.6814 |
| Mammary tumor | 21 | *Bcap31, Sp2, Ptdss1, Snx4, Cript, Ppp6c, Ak1, Cstf1, Mme, Polr2b, Nfyc, Vwa9, Ovol2, Dido1, Lap3, Pglc3, Fbxo28, Mest, Serpinb6a, Morf4l1, Tut1* | 0.8528 |
| Hippocampus | 11 | *Sod2, Vcl, Ak1, Mme, Capz, Psmc2, Actb, Mras, Tubb2b, Cfl1, Rnf169* | 0.8071 |
| Embryonic germ cell | 3 | *Dennd2c, Mme, Zfp936* | 0.7612 |
